# Exposure to Inorganic Arsenic Is Associated with Increased Mitochondrial DNA Copy Number and Longer Telomere Length in Peripheral Blood

**DOI:** 10.3389/fcell.2016.00087

**Published:** 2016-08-22

**Authors:** Syeda S. Ameer, YiYi Xu, Karin Engström, Huiqi Li, Pia Tallving, Barbro Nermell, Analia Boemo, Luis A. Parada, Lidia G. Peñaloza, Gabriela Concha, Florencia Harari, Marie Vahter, Karin Broberg

**Affiliations:** ^1^Department of Laboratory Medicine, Division of Occupational and Environmental Medicine, Lund UniversityLund, Sweden; ^2^Unit of Metals and Health, Institute of Environmental Medicine, Karolinska InstitutetStockholm, Sweden; ^3^Facultad de Ciencias Exactas and Consejo de Investigación, Universidad Nacional de SaltaSalta, Argentina; ^4^Institute of Experimental Pathology – UNSa – CONICETSalta, Argentina; ^5^Risk Benefit Assessment Unit, Science Department, National Food AgencyUppsala, Sweden

**Keywords:** MMA, energy, oxidative phosphorylation, cancer, AS3MT

## Abstract

**Background:** Exposure to inorganic arsenic (iAs) through drinking water causes cancer. Alterations in mitochondrial DNA copy number (mtDNAcn) and telomere length in blood have been associated with cancer risk. We elucidated if arsenic exposure alters mtDNAcn and telomere length in individuals with different arsenic metabolizing capacity.

**Methods:** We studied two groups in the Salta province, Argentina, one in the Puna area of the Andes (*N* = 264, 89% females) and one in Chaco (*N* = 169, 75% females). We assessed arsenic exposure as the sum of arsenic metabolites [iAs, methylarsonic acid (MMA), dimethylarsinic acid (DMA)] in urine (U-As) using high-performance liquid chromatography coupled with hydride generation and inductively coupled plasma mass spectrometry. Efficiency of arsenic metabolism was expressed as percentage of urinary metabolites. MtDNAcn and telomere length were determined in blood by real-time PCR.

**Results:** Median U-As was 196 (5–95 percentile: 21–537) μg/L in Andes and 80 (5–95 percentile: 15–1637) μg/L in Chaco. The latter study group had less-efficient metabolism, with higher %iAs and %MMA in urine compared with the Andean group. U-As was significantly associated with increased mtDNAcn (log2 transformed to improve linearity) in Chaco (β = 0.027 per 100 μg/L, *p* = 0.0085; adjusted for age and sex), but not in Andes (β = 0.025, *p* = 0.24). U-As was also associated with longer telomere length in Chaco (β = 0.016, *p* = 0.0066) and Andes (β = 0.0075, *p* = 0.029). In both populations, individuals with above median %iAs showed significantly higher mtDNAcn and telomere length compared with individuals with below median %iAs.

**Conclusions:** Arsenic was associated with increased mtDNAcn and telomere length, particularly in individuals with less-efficient arsenic metabolism, a group who may have increased risk for arsenic-related cancer.

## Introduction

Elevated arsenic concentrations in drinking water occur in many countries, e.g., in Argentina, Bangladesh, India, and some parts of the U.S.A. Arsenic exposure is a known risk factor for different types of malignancies, mainly of epithelial origin, such as cancer of the lung, bladder, kidney, skin, liver, and prostate (International Agency for Reseasrch on Cancer, 2012). However, the mechanisms behind the carcinogenic effect of arsenic have not been completely clarified.

The susceptibility to arsenic differs between individuals and a key factor for individual susceptibility is the capacity to metabolize arsenic and excrete it efficiently. Inorganic arsenic (iAs) is metabolized to methylarsonic acid (MMA), and further, to dimethylarsinic acid (DMA). In humans, like most mammals, efficient methylation from iAs to DMA is associated with decreased reactivity and increased rate of urinary arsenic excretion (Vahter, 2002; Gardner et al., 2011). The degree of methylation to DMA varies within and between individuals and populations suggesting a genetic component for the arsenic metabolism (Vahter, 2002). Incomplete arsenic metabolism seems to be a marker of increased susceptibility to arsenic-related diseases: individuals with high fraction of inorganic arsenic and/or MMA in urine have higher risk for skin lesions and skin cancer (Lindberg et al., 2008; Pilsner et al., 2009; Antonelli et al., 2014; Engström et al., 2015) and cardiovascular disease (Chen et al., 2013; Wu et al., 2015) compared to individuals with low fraction of inorganic arsenic and/or MMA in urine. Genetic variation in arsenite methyltransferase (*AS3MT*), which encodes the major arsenic-methylating enzyme, significantly contributes to arsenic metabolism efficiency (Engström et al., 2011; Schlebusch et al., 2013, 2015) and is likely an underlying factor partly determining variation in susceptibility to arsenic between individuals and populations (Hossain et al., 2012; Pierce et al., 2013; Engström et al., 2015).

Telomeres, the tandem repeats (TTAGGG) at the ends of the chromosomes, protect the chromosomes from recombination and degradation (Blasco, 2005), which could lead to genome instability. In cross-sectional and prospective studies, alterations in telomere length in peripheral blood have been associated with increased risk for several types of cancer (Ma et al., 2011), such as cancers in the head and neck, bladder, breast, skin and lung (Wu et al., 2003; McGrath et al., 2007; Shen et al., 2007, 2011; Han et al., 2009; Seow et al., 2014). Previously, we (Li et al., 2012) and others (Zhang et al., 2013) reported that arsenic in urine was associated with longer telomeres in peripheral blood suggesting that arsenic modifies the individual telomere length.

Another cancer-related genetic component is the mitochondrial DNA (mtDNA). The mitochondrion is the key organelle for energy metabolism, but it also has important functions in the regulation of apoptosis (Lee and Wei, 2000) and cell proliferation (Martinez-Outschoorn et al., 2011). Recent findings have suggested that mitochondrial metabolic disease can lead to cancer (Seyfried, 2015). Mitochondria possess their own extra-nuclear circular DNA that exists in different copy numbers (mtDNAcn) in each mitochondrion. Mitochondria generate high levels of reactive oxygen species (ROS) during energy production. Lack of introns and protective histones and its proximity to the electron transport chain make the mtDNA highly susceptible to oxidative damage (Van Houten et al., 2006). Alterations in mtDNAcn may be associated with cancer risk as indicated by case-control and prospective cohort studies which have shown that mtDNAcn is associated with increased risk of cancer in the breast, head and neck, prostate, kidney and colon/rectum in women (Thyagarajan et al., 2013; Cheau-Feng Lin et al., 2014; Huang et al., 2014; Zhou et al., 2014; Lemnrau et al., 2015; Melkonian et al., 2015).

We have previously reported that the indigenous people living in the Andean part of the Salta province in the northern Argentina, the so called Puna area, have a unique capacity of metabolizing inorganic arsenic, mainly due to presence of genetic polymorphisms in *AS3MT*, which is one of the main genes regulating arsenic methylation (Vahter et al., 1995; Engström et al., 2011; Schlebusch et al., 2013, 2015). Other areas in Salta province, such as Chaco, situated in the eastern part of the Salta province, also have arsenic-contaminated drinking water (Concha et al., 1998). People living in Chaco are mainly of mixed ethnicity (descendants of Spanish/European). The local health authority in Salta invited our group to conduct a study there with the aim to compare the populations from the Andes and the Chaco regions for arsenic metabolism and toxicity. Thus, the aim of the present study was to evaluate the effect of arsenic, including modifying effects of the arsenic metabolizing capacity, on the cancer-related markers telomere length and mtDNAcn in two arsenic-exposed study groups with different genetic background in northern Argentina.

## Materials and methods

### Study areas and study participants

#### Andes

Two hundred and sixty four individuals, including men (*n* = 28) and women (*n* = 236), were recruited in 2008 (*n* = 218) and 2011 (*n* = 46) in the Puna area of the Andes Mountains (about 3800 m above sea level) in the province of Salta, northern Argentina. The recruitments in 2008 and 2011 were performed in a similar way and individuals who participated in 2008 were not recruited in 2011. Most of the individuals were living in the village of San Antonio de los Cobres (*n* = 192), which has elevated arsenic concentrations in the drinking water (~200 μg/L), and 72 individuals were living in small surrounding villages with lower water arsenic concentrations (3.5–70 μg/L) (Concha et al., 2010). The source of drinking water in San Antonio de los Cobres is a natural spring, Agua de Castilla, located about 1 km outside the village, and the water is chlorinated before being distributed through pipes to the village (Concha et al., 2010). The total population of San Antonio de los Cobres and surrounding villages is about 8100 inhabitants, and are mainly of indigenous origin (Kolla ethnicity).

Details of the recruitments have been described elsewhere (Engström et al., 2011). Briefly, individuals were interviewed about their age, ancestry, time of residence, drinking water sources, dietary habits, medical history, smoking, alcohol consumption, coca chewing, as well as parity (for women). Also, weight and height were measured and used for calculation of body mass index (BMI). We have previously reported a positive association of arsenic exposure with telomere length in the women recruited from this study area in 2008 (Li et al., 2012).

#### Chaco

One hundred and sixty-nine individuals were recruited in the Chaco and Anta areas east of the city of Salta (up to 300 m above sea level); here referred to as participants from Chaco. Individuals in Chaco were informed about the study by radio announcements, by primary health care workers, and by contacting individuals in the lines at the post-office. In the Rivadavia hospital the patients' attendants were asked to participate and those who wanted to volunteer were included in the study. Individuals were living in different areas [El Rincón (*n* = 25), Los Rosales (*n* = 22) and other villages in rural Anta county (*n* = 36); Rivadavia (*n* = 26), La Unión (*n* = 12) and Nueva Población (*n* = 6) in Rivadavia county; and El Galpón (*n* = 42) in Metán county]. The recruitment was performed in 1999 (Anta) and 2013 (all other areas). The arsenic concentrations in drinking water (mainly well water) varied between 110 and 837 μg/L in Anta (several water sources), 4.7 μg/L in El Galpón (samples collected from several small villages), about 230 μg/L in Rivadavia, 72 μg/L in Los Rosales, 983 μg/L in Nueva Población and 23 μg/L in La Unión. The total population in Anta is ~8000 inhabitants, in El Galpón ~8400, and in Rivadavia, and La Unión 6400. The people are mostly “creoles,” i.e., of Spanish/European descents. Sampling was done following the standard procedure developed by Unit of Metals and Health, Institute of Environmental Medicine at Karolinska Institutet, and the study participants in Chaco were asked similar questions as in the Andes.

Men and women, 16 years and older, were included in the study. The Health Ministry of Salta (Salta, Argentina) and the Regional Ethical Committee at the Karolinska Institutet (Stockholm, Sweden) approved the study. Oral and written informed consent was obtained from all study participants.

### Urine and blood sampling

Spot urine and blood samples were collected from all study participants and the sample collection was performed throughout the day. Mid-stream urine samples were collected in plastic urine collection cups and then transferred to 24 mL polyethylene tubes and immediately frozen. Peripheral blood samples were collected in K_2_EDTA tubes (Vacuette; Greiner Bio-One, Greiner, Germany) for DNA isolation.

### Analysis of arsenic exposure

We measured total arsenic concentrations in water samples collected from the Andes and Chaco regions by inductively coupled plasma mass spectrometry (ICPMS: Agilent 7500ce; Agilent Technologies, Tokyo, Japan) as previously described (Concha et al., 2010). Water from rural Anta was analyzed by atomic absorption spectrophotometry (AAS) (Concha et al., 2006). We did not perform speciation of arsenic in the water. Most of the absorbed pentavalent arsenic is rapidly reduced in blood (Vahter, 2002), before the trivalent form (but not the pentavalent) is taken up by the hepatocytes where it is methylated (Lerman et al., 1983). Exposure to arsenic was determined based on the sum of concentrations of iAs and its metabolites (MMA + DMA) in urine (U-As). We assessed the efficiency of arsenic metabolism by the fractions (percentages) of iAs, MMA, and DMA in urine (Vahter, 2002). Arsenic metabolites in urine were measured by high-performance liquid chromatography (HPLC: Agilent 1100 series system, Agilent Technologies, Waldbronn, Germany) coupled with hydride generation (HG) and ICPMS (HPLC-HG-ICPMS) as described previously (Gardner et al., 2011). Urine samples from rural Anta was analyzed for arsenic metabolites by HPLC-AAS, as previously described (Concha et al., 2006). To compensate for variation in dilution of urine, the arsenic concentrations were adjusted to the mean specific gravity, measured by a hand refractometer (Atago, Japan) or a digital refractometer (EUROMEX RD 712 clinical refractometer; EUROMEX, Arnhem, the Netherlands). We evaluated the data using overall specific gravity value of 1.020 for adjustment for all samples.

### Analysis of mitochondrial DNA copy number

Human mtDNA is a small, double-stranded DNA encoding 22 tRNAs, 2 rRNAs, and 13 genes that code for protein subunits of the electron transport chain complexes. The primers were selected from the mtDNA tRNA region, because other mtDNA regions are highly polymorphic and may contain common deletions. The mitochondrial tRNA^Leu (UUR)^ gene region is rarely deleted and contains only a few rare single nucleotide polymorphisms (mtSNP) (Venegas and Halberg, 2012). The relative mtDNAcn was measured based on the method by Hou et al. (2010) by using real-time PCR (7900HT, Applied Biosystems, Foster City, CA, U.S.A.). Master mixes were prepared with KAPA SYBR FAST qPCR Kit Master Mix (2X) ABI Prism (Kapa Biosystems, Woburn, MA, U.S.A.), and primers (0.20 μM for each primer) for mtDNAcn were: forward 5′-CAC CCA AGA ACA GGG TTT GT-3′ and reverse 5′-TGG CCA TGG GTA TGT TGT TA-3′; and primers for the hemoglobin beta (*HBB*) gene were: forward 5′-TGT GCT GGC CCA TCA CTT TG-3′ and reverse 5′-ACC AGC CAC CAC TTT CTG ATA GG-3′. The mtDNA amplicon is 107 bp long and the mtDNA primers are specific to mtDNA as evaluated by BLAST (https://blast.ncbi.nlm.nih.gov/Blast.cgi)(Gen bank accession number: Accession no: NC 012920.1). Each reaction (end volume 10 μl) consisted of 2.5 μl of DNA (4 ng/μl) and 7.5 μl master mix. The thermal cycle profile was 95°C for 3 min, followed by 95°C for 3 s and 60°C for 20 s for 25 cycles (mtDNAcn) or 35 cycles (*HBB*). A standard curve, one control sample, and a sample with no template were also included in each run. All samples and standard curve points were run in triplicate. For the standard curve, one reference DNA sample was diluted serially twofold to produce five concentrations of 1–16 ng/μl. *R*^2^ for each standard curve was >0.99. Standard deviations of < 0.1 were accepted for the *C*_t_ values of each triplicate. SDS 2.4.1 software (Life Technologies) calculated the relative quantity of mtDNAcn and *HBB* for each sample, based on the standard curve. The relative mtDNAcn was the quotient of the quantity of mtDNAcn and *HBB*, and thus, it is an arbitrary value. The coefficient of variation (CV) of the control sample included in every run was 12%.

### Analysis of telomere length

DNA was isolated from peripheral blood with the Qiagen DNA blood Midi kit (Qiagen, Hilden, Germany). The relative telomere length was measured using real-time PCR (7900HT), as described previously (Li et al., 2012). Briefly, the master mix for telomere length determination was prepared with telomere-specific primers (0.45 μM of each primer), 1 × PCR Buffer (all PCR reagents from Life Technologies) 1.75 mM MgCl_2_, 0.8 mM dNTPs, 0.3 mM SYBR Green, 1 × Rox, and 0.5 U Platinium *Taq*. Master mix for *HBB* runs were prepared with *HBB* primers (0.40 μM for each primer) and KAPA SYBR FAST qPCR Kit Master Mix (2X) ABI Prism (Kapa Biosystems). Five microliters of sample DNA (4 ng/μl) was added to each reaction (final volume 20 μl). A standard curve, a reference DNA, and a negative control were also included in each run, all samples, standards, and controls were run in triplicate. For the standard curve, one calibrator DNA sample was diluted serially by twofold, to produce concentrations of 1–16 ng/μl. R^2^ for each standard curve was >0.99. Standard deviations (for *C*_*t*_ values of each triplicate) were accepted at < 0.1. The relative length of the telomeres was obtained through calculating the ratio (T/S) of the telomere repeat product to a single-copy gene product (S, here *HBB*) for each individual, by the formula T/S = 2^−ΔCt^, where ΔCt = Ct_telomere_ − Ct_HBB_. This ratio was then compared with the ratio of the reference DNA. The telomere length ratio is an arbitrary value. The CV of the reference DNA included in every run was 9.8%. The telomere length for subjects in the Andes and Chaco were measured at two different time-points and with different reference DNAs. To make the data comparable, we measured the ratio between the two different reference DNAs and adjusted the values of Andes by this ratio, so that all data were based on the same reference DNA.

### Statistical analyses

Statistical analyses were performed using SPSS software, version 22 (IBM, Chicago, IL, USA). Statistical significance refers to *p* < 0.05. Data from the study groups in the Andes and Chaco were analyzed separately.

Mann-Whitney U test was used to evaluate the differences in characteristics of the study participants between the study groups. In order to investigate how the characteristics differed with U-As, we stratified U-As into tertiles to evaluate potential non-linear differences in characteristics by Jonckheere-Terpstra trend test.

We evaluated the associations between U-As, mtDNAcn, telomere length and potentially confounders (age, gender, BMI, and chewing of coca leaves) using Spearman's rank correlation coefficient. The linearity between U-As and mtDNAcn as well as U-As and telomere length were inspected using scatter plots. In order to improve linearity between U-As and mtDNAcn, we log2-transformed mtDNAcn for both Andes and Chaco. Linear regression analyses were performed to evaluate the associations between U-As and log2-mtDNAcn, and U-As and telomere length. The crude model (model 1) included only U-As and the adjusted model (model 2) included age and gender as covariates (confounders). Age and gender were selected as covariates based on the knowledge from published literature. Other covariates such as BMI and coca chewing were considered for adjustments in sensitivity analyses (see below).

The influence of efficiency of arsenic metabolism on the associations between U-As and log2-mtDNAcn or U-As and telomere length were analyzed by including an interaction term between U-As and the fraction of each metabolite separately (defined as above and below median of %iAs, %MMA, or %DMA). We also performed linear regression analyses with individuals stratified by median values of the U-As metabolites.

Several sensitivity analyses were performed. The differences in telomere length or mtDNAcn could reflect alterations in cell composition. We therefore analyzed the associations between U-As and log2-mtDNAcn as well as U-As and telomere length in a subgroup (*n* = 79 Andean women) where we adjusted for estimated fractions of cell types (CD4 and CD8 T-cells, B-cells, natural killer cells, monocytes, and granulocytes) in blood. The cell composition was derived from epigenetic signatures of cells retrieved from whole-genome DNA methylation data using Infinium HumanMethylation 450 K BeadChip [lllumina, CA, U.S.A., Engström et al., 2013] and was estimated by the method by Houseman et al. (2012), which estimates the relative proportion of pure cell types within a sample, where the DNA methylation signature is a surrogate for the distribution of white blood cells. We also did sensitivity analysis for chewing of coca leaves in the Andes only, since none of the participants chewed coca-leaves in Chaco. We had information about smoking and BMI for a few individuals in Chaco and mainly from individuals with low exposure to arsenic; therefore sensitivity analyses with adjustments for smoking or BMI were not considered meaningful. Further, few individuals (*n* = 6) in Chaco reported hyperkeratosis, we also performed sensitivity analyses excluding those individuals to see the effect of arsenic on mtDNAcn and telomere length in rest of the Chaco population.

## Results

### Characteristics of the study groups

The general characteristics of the two study groups are presented in Table 1 (characteristics stratified by tertiles of U-As are presented in Supplementary Material Table S2) and the drinking water concentrations in the different villages are presented in Supplementary Material Table S1. The arsenic concentrations in water showed a large range both in Andes and Chaco and the corresponding U-As concentrations indicated that the major exposure source to arsenic was the drinking water (Andes: *r*_S_ = 0.72, *p* < 0.001; Chaco: *r*_S_ = 0.87, *p* < 0.001). The study groups differed in height and weight, where individuals in Andes were shorter and weighed less than in Chaco. Most participants were women; 11% in Andes and 25% in Chaco were men. We observed a trend of increase in BMI by increasing U-As concentrations in Chaco (Supplementary Material Table S2). The overall median U-As was 196 μg/L and 80 μg/L for the Andes and Chaco study groups, respectively. The metabolite pattern differed significantly between the study groups: the median % iAs and %MMA were higher and the median %DMA was lower in Chaco compared with the Andes (Table 1). The %iAs increased and %DMA decreased significantly with increasing U-As tertiles in the Andes, and %MMA increased and %DMA decreased significantly with increasing U-As tertiles in Chaco (Supplementary Material Table S2).

**Table 1:** **Characteristics of the Andes and Chaco study groups**.

	**Andes**			**Chaco**			
**Variable**	***n***	**Median**	**5/95 percentile**	***n***	**Median**	**5/95 percentile**	***p*-value ^a^**
Age (years)	264	35	20 to 65	169	39	18 to 69	0.078
Sex	264		89% female	169		75% female	< 0.001
Height (cm)	264	153	144 to 167	127	158	148 to 174	< 0.001
Weight (kg)	264	59	45 to 84	127	63	43 to 92	0.023
BMI (kg/m^2^)	264	25	19 to 35	127	25	18 to 36	0.80
Parity	243	3	0 to 11			No data	
Arsenic in urine (μg/L)	264	196	21 to 537	169	80	15 to 1637	< 0.001
iAs in urine (%)	264	11	4.3 to 23	169	17	5.6 to 34	< 0.001
MMA in urine (%)	264	7.9	4.0 to 14	169	10	3.0 to 22	< 0.001
DMA in urine (%)	264	81	66 to 91	169	71	49 to 86	< 0.001
mtDNAcn^b^	244	0.57	0.33 to 1.0	159	0.84	0.44 to 1.5	< 0.001
Telomere length^b^	208	0.43	0.30 to 0.58	159	1.10	0.73 to 1.7	< 0.001

a p-value is derived from the Mann-Whitney U-test.

b The relative mtDNAcn/telomere length was the quotient of the quantity of mtDNAcn/telomere and HBB, and thus, it is an arbitrary value.

The median values of relative mtDNAcn and the relative telomere length differed significantly between the study groups, where participants in Chaco had higher mtDNAcn and telomere length than participants in Andes (*p* < 0.001, Table 1). The median values of mtDNAcn and telomere length differed significantly between the U-As tertiles in Chaco (Supplementary Material Table S2). MtDNAcn and telomere length were positively correlated (Andes, *r*_S_ = 0.14, *p* = 0.054; Chaco, *r*_S_ = 0.29, *p* < 0.001). Telomere length was negatively correlated with age in both study groups (Ande*s r*_S_ = −0.31, *p* < 0.001, Chaco *r*_S_ = −0.36, *p* < 0.001), while mtDNAcn was not correlated with age in any of the groups. The median mtDNAcn differed between the sexes; it was lower for men in Andes but lower for women in Chaco (Andes: women = 0.58, men = 0.54, *p* = 0.15; Chaco: women = 0.77, men = 0.98, *p* = 0.001). The median telomere length was higher for men in Chaco, but it did not differ between the sexes in the Andes (Andes: women = 0.43, men = 0.43, *p* = 0.29; Chaco: women = 1.06, men = 1.21, *p* = 0.031).

The study group from Chaco included six study participants in a family clinically diagnosed with arsenic related hyperkeratosis. These six participants had very high U-As concentrations (median 1943 μg/L; range 623–2258 μg/L) and also high %MMA in urine (average 21%; range 18–24%) compared with the other study participants in Chaco (Supplementary Material Table S3). Their telomere length did not differ from the rest of the participants in Chaco, but their mtDNAcn was higher (median relative values 1.1 vs. 0.83).

### Arsenic exposure, mitochondrial DNA copy number, and telomere length

**Figure 1:**
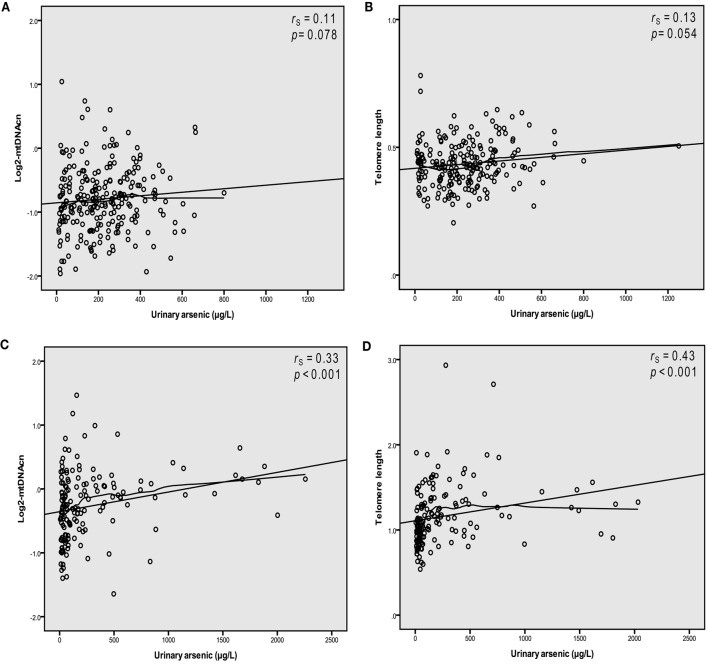
**Arsenic in urine is associated with higher copy number of mitochondrial DNA (log2-transformed, abbreviation mtDNAcn) and longer telomeres in blood**. Scatter plots depicting relations between urinary arsenic (sum of inorganic arsenic metabolites) concentrations and log2-mtDNAcn and telomere length in Andes **(A,B)** and Chaco **(C,D)**.

We observed linear relationships between U-As and log2-mtDNAcn, as well as U-As and telomere length in blood in both study groups (Figure 1). Increasing U-As was significantly associated with both higher log2-mtDNAcn and with longer telomere length in Chaco, and to a lesser extent for both outcomes in Andes (models 1 and 2; Table 2). Among the Andean women, we made further adjustments for estimated cell proportions of white blood cells (CD4 and CD8 T-cells, B-cells, natural killer cells, monocytes, and granulocytes). MtDNAcn and telomere length were not significantly correlated with the estimated cell proportion for any cell type. When adjusting the analyses for estimated cell proportions, the effect estimates for U-As decreased between 9 and 10% for log2-mtDNAcn and about 2% for telomere length, but the associations still remained with *p* < 0.05. We also adjusted the analyses for chewing of coca-leaves in the Andes: the effect estimates for U-As did not change for mtDNAcn, but increased 3% for telomere length. Finally, we excluded people with hyperkeratosis in Chaco, and we found that the association between U-As and mtDNAcn or U-As and telomere length were not driven by these individuals, and the associations still remained below *p* = 0.05.

**Table 2:** **Associations of U-As^a^ with log2-mtDNAcn and telomere length in peripheral blood**.

**Marker/study group**	**Model 1^b^**			**Model 2^c^**	
	***n***	**β (95% CI)**	***p*-value**	**β (95% CI)**	***p*-value**
**mtDNAcn**					
Andes	244	0.028 (−0.014 to 0.069)	0.19	0.025 (−0.017 to 0.066)	0.24
Chaco	159	0.031 (0.011 to 0.050)	0.0024	0.027 (0.0069 to 0.046)	0.0085
**TELOMERE LENGTH**					
Andes	208	0.0071 (0.00012 to 0.014)	0.046	0.0075 (0.00078 to 0.014)	0.029
Chaco	159	0.019 (0.0070 to 0.032)	0.0024	0.016 (0.0045 to 0.028)	0.0066

a U-As referred to as sum of urinary arsenic metabolites.

b Log2-mtDNAcn/telomere length = α + β1 × U-As (per 100 μg/L).

c Log2-mtDNAcn/telomere length = α + β1 × U-As (per 100 μg/L) + β2 × age + β3 × gender.

### Modification by arsenic metabolism efficiency

Because the arsenic metabolism efficiency has been shown to be a susceptibility factor for arsenic-related disease (Antonelli et al., 2014), we further evaluated the role of the arsenic metabolism for effects of arsenic on telomere length and mtDNAcn. We found statistically significant interactions between %iAs and U-As, as well as between %DMA and U-As on the effect of log2-mtDNAcn in the Andes (adjusted models, *p* = 0.013 for %iAs, *p* = 0.0083 for %DMA), but not in Chaco (Table 3). After stratifying the data sets at the median values of %iAs and %DMA, there were, both in Andes and Chaco, significant positive associations between U-As and log2-mtDNAcn among individuals with high %iAs and low %DMA, but not among individuals with low %iAs and high %DMA.

**Table 3:** **Multivariable regression analyses of the associations between U-As and log2-mtDNAcn stratified for arsenic metabolism efficiency (above and below median of fraction of each metabolite)^a^**.

**Study group**	**mtDNAcn^b^**	**% iAs**	***n***	**β_1_ (95% CI)^c^**	***p*-value^c^**	***p*-value^d^**
Andes	0.56	< 11	122	−0.018 (−0.076 to 0.039)	0.53	0.013
	0.58	≥11	122	0.084 (0.022 to 0.15)	0.0080	
Chaco	0.76	< 17	80	0.012 (−0.020 to 0.044)	0.45	0.82
	0.93	>17	79	0.028 (0.0019 to 0.054)	0.036	
		**% DMA**				
Andes	0.58	< 80.9	122	0.076 (0.017 to 0.13)	0.011	0.0083
	0.56	≥80.9	122	−0.030 (−0.092 to 0.031)	0.33	
Chaco	0.94	< 71.1	79	0.027 (0.0038 to 0.049)	0.023	0.83
	0.77	≥71.1	80	0.020 (−0.038 to 0.079)	0.49	

a U-As referred to as sum of urinary arsenic metabolites.

b Presented as median relative values of mtDNAcn.

c Log2-mtDNAcn = α + β1 × U-As (per 100 μg/L) + β2 × age + β3 × gender.

d p-value for interaction (β4) from the equation: Log2-mtDNAcn = α + β1 × U-As (per 100 μg/L) + β2 × age + β3 × gender + β4 × (U-As × < and > median %iAs/%MMA/%DMA).

There were no significant interactions between fractions of arsenic metabolites and U-As on telomere length (Table 4). However, in the stratified analyses, statistically significant positive associations between U-As and telomere length were only found among individuals with high %iAs in both the study groups. There was less of a clear pattern for modification by %DMA or by %MMA (Supplementary Material Table S4).

**Table 4:** **Multivariable regression analyses of the associations between U-As and telomere length stratified for arsenic metabolism efficiency (above and below median of fraction of each metabolite)^a^**.

**Study group**	**TL^b^**	**% iAs**	***n***	**β_1_ (95% CI)^c^**	***p*-value^c^**	***p*-value^d^**
Andes	0.41	< 11	94	0.0024 (−0.0089 to 0.014)	0.67	0.35
	0.45	≥11	114	0.0089 (0.00036 to 0.017)	0.041	
Chaco	1.01	< 17	80	0.011 (−0.0059 to 0.028)	0.20	0.35
	1.19	>17	79	0.018 (0.0019 to 0.034)	0.029	
		**% DMA**				
Andes	0.45	< 80.9	114	0.0069 (−0.0012 to 0.015)	0.093	0.73
	0.41	≥80.9	94	0.0051 (−0.0076 to 0.018)	0.43	
Chaco	1.23	< 71.1	79	0.012 (−0.0028 to 0.026)	0.11	0.61
	1.001	≥71.1	80	0.024 (−0.0053 to 0.054)	0.11	

a U-As referred to as sum of urinary arsenic metabolites. The number of individuals in Andes in each < and ≥ median split groups varied for telomere length as telomere length measurements were only available for individuals sampled in 2008.

b Presented as median relative values of telomere length.

c Telomere length = α + β1 × U-As (per 100 μg/L) + β2 × age + β3 × gender.

d p-value for interaction (β4) from the equation: telomere length = α + β1 × U-As (per 100 μg/L) + β2 × age + β3 × gender + β4 × (U-As × < and > median %iAs/%MMA/%DMA).

## Discussion

In this study, we explored associations of arsenic exposure through drinking water, assessed by the concentration of arsenic metabolites in urine, with carcinogenic markers (telomere length and mtDNAcn) in two study groups with different ethnic backgrounds. We found that the effect of arsenic exposure varied between the study groups. In Chaco, arsenic exposure showed persuasive associations with both telomere length and mtDNAcn, but this was not so clear in the Andes, where only association was found with telomere length. This discordance in effects likely relates to variation in the efficiency of arsenic metabolism: the Chaco individuals had, compared with the Andes, in general less-efficient metabolism, reflected by higher fractions of iAs and MMA and lower fractions of DMA in urine. Associations between U-As and the DNA markers were observed also in the Andean study group, but only in the individuals with less efficient metabolism, i.e., with highest fraction of iAs in urine. These findings stress the role of arsenic metabolism in the toxicity of arsenic, and that some individuals and population groups, due to their lower capacity for arsenic biotransformation, might be more susceptible to arsenic toxicity than others. We recently reported a signal for genetic selection in the *AS3MT* gene in people in the Andes (Schlebusch et al., 2013, 2015), partly overlapping with the present study group, and our data here support our hypothesis that the indigenous people in the Andes show fewer adverse effects of arsenic exposure due to a long-term adaptation to an arsenic-rich environment, compared with a study group like the Chaco, where people have lived in an arsenic-contaminated area for much shorter time.

Similar to our study, others have found that blood mtDNAcn increased in response to exposure to different carcinogenic hazards, such as exposure to low levels of benzene, maternal smoking, and sun exposure (Carugno et al., 2012; Gebhard et al., 2014; Stangenberg et al., 2015), but there are only few studies addressing the influence of arsenic on mtDNAcn. One study reported increased mitochondrial biogenesis and function in arsenic-induced skin cancer patients compared with healthy subjects (Lee et al., 2011). In contrast, human-hamster AL cells treated with high concentrations of sodium arsenite (0.5–1 mg/L), showed decreased mtDNAcn along with increased large heteroplasmic mtDNA deletions (Partridge et al., 2007). It has been suggested that the increased oxidative stress from exposure to toxic compounds has a dual influence on the mtDNA (Hou et al., 2010). Mild stress might stimulate mtDNA synthesis and increase the number of mitochondria to fulfill the need for cellular respiratory capacity for cell survival. However, excessive oxidative stress may cause decreased mtDNA synthesis, possibly due to increasing defects in mitochondria resulting in apoptosis and cell death (Hou et al., 2010). This duality in response to mild vs. excessive oxidative stress might also explain previous findings on the effects of arsenic on telomere length. In human cord blood cells, sub-nM arsenite increased *TERT* (the main telomere-maintaining enzyme) gene and protein expression *in vitro*, resulting in maintained telomere length, while at 1 μM arsenite, *TERT* expression and telomere length decreased (Ferrario et al., 2009). In people exposed to arsenic via drinking water (1–1000 μg/L) in Inner Mongolia, *TERT* expression was positively associated with arsenic concentrations in water and in fingernails, and with the severity of arsenic related hyperkeratosis (Mo et al., 2009). The latter should be noted as we found a family in Chaco demonstrating hyperkeratosis. This family did not have longer telomeres, but did have more mtDNAcn compared with the individuals without hyperkeratosis in Chaco. These findings, however, should be very cautiously interpreted as they were based on few study subjects.

We found a significant correlation between telomere length and mtDNAcn in both study groups. *TERT* maintains telomere length under oxidative stress, but in a time- and dose-dependent manner, *TERT* gets excluded from the nucleus and localizes in the mitochondria, giving protection by decreasing mitochondrial superoxide production and cell peroxide levels (Ahmed et al., 2008). One can hypothesize that in our study with low to moderate exposure to arsenic, arsenic stimulates *TERT* expression, which in turn results in telomere lengthening as well as stimulation of mitochondrial DNA production.

However, in prospective studies, longer telomeres in blood have been associated with increased risk for lung (Shen et al., 2011; Seow et al., 2014) and pancreatic cancers (Lynch et al., 2013). We found in both study groups that arsenic was associated with longer telomeres in blood, even after taking cell profiles in blood into account. This confirms earlier findings (Ferrario et al., 2009) and provides strong evidence that arsenic has a true positive effect on telomeres. Arsenic causes lung cancer (International Agency for Reseasrch on Cancer, 2012), and our findings here suggests a mechanistic link that this occurs by lengthening of the telomeres. Higher mtDNAcn in blood has in one prospective study been associated with increased risk of breast cancer (Thyagarajan et al., 2013; Lemnrau et al., 2015), and in case-control studies been linked to head and neck cancer (Cheau-Feng Lin et al., 2014), prostate cancer (Zhou et al., 2014), and chronic lymphocytic lymphoma (Hosnijeh et al., 2014). Of these cancer forms, arsenic has to some extent been associated with breast and prostate cancer (López-Carrillo et al., 2014; Bardach et al., 2015). MtDNAcn seems to be a promising marker for future cancer risk, but these women need to be followed up in order to evaluate the actual link between arsenic, mtDNAcn, telomere length and cancer.

Telomere length and mtDNAcn levels differed between the study groups, where the Andean study group showed much lower median values of mtDNAcn and telomere length compared with the Chaco study group. We speculate that this disparity is related to their ethnic backgrounds and living conditions, particularly the differences in altitude. High altitude is correlated with high hemoglobin (Ameer et al., 2015), and in turn high iron levels in blood. Iron concentrations is associated with increased oxidative stress (Tuomainen et al., 2007), which could shorten the telomeres and affect the mtDNA contents.

In conclusion, we here provide evidence that arsenic exposure through drinking water increases the telomere length and mtDNAcn, two pre-carcinogenic markers, and that the arsenic methylating capacity modifies the degree of DNA changes.

## Author contributions

KB and MV conceived the study. KE, PT, AB, LAP, LGP, GC, FH, BN, MV, and KB did the sampling and data collection. GC, BN, FH, and MV performed the arsenic measurements. SSA, YX, and HL designed the experiments for mtDNA and TL and performed them. SSA performed statistical analysis, interpreted the data. SSA and KB wrote the manuscript, which was reviewed by the coauthors. All authors read and approved the final version of the manuscript.

### Conflict of interest statement

The authors declare that the research was conducted in the absence of any commercial or financial relationships that could be construed as a potential conflict of interest.
