# Supplementary material for: Exposure to Inorganic Arsenic Is Associated with Increased Mitochondrial DNA Copy Number and Longer Telomere Length in Peripheral Blood
Source: Front Cell Dev Biol. 2016 Aug 22;4:87. doi: 10.3389/fcell.2016.00087 (PMC4992680; doi:10.3389/fcell.2016.00087)
Supplement: Supplementary file 1 [file Table1.DOCX]

Supplementary Material

Article Title

**Exposure to inorganic arsenic is associated with increased mitochondrial DNA copy number and longer telomere length in peripheral blood**

Syeda Shegufta Ameer^1^, YiYi Xu^1^, Karin Engström^2^, Huiqi Li^1^, Pia Tallving^1^, Barbro Nermell^2^, Analia Boemo^3^, Luis A. Parada^4^, Lidia Guadalupe Peñaloza^3^, Gabriela Concha^5^, Florencia Harari^2^, Marie Vahter^2^, Karin Broberg^2*^

*1) Department of Laboratory Medicine, Division of Occupational and Environmental Medicine, Lund University, Lund, Sweden*

*2) Institute of Environmental Medicine, Unit of Metals & Health, Karolinska Institutet, Stockholm, Sweden*

*3) Facultad de Ciencias Exactas & Consejo de Investiga ción, Universidad Nacional de Salta, Salta, Argentina*

*4) Institute of Experimental Pathology – UNSa – CONICET, Salta, Argentina*

*5) Science Department, Risk Benefit Assessment Unit, National Food Agency, Uppsala, Sweden*

^*^ Corresponding author

Address correspondence to Karin Broberg, Institute of Environmental Medicine, Unit of Metals & Health, Karolinska Institutet, Stockholm, Sweden. Telephone: +46737823750. Fax: +46 08336981; E-mail: [karin.broberg@ki.se](mailto:karin.broberg@ki.se)

**Supplementary Material Table S1:** Concentrations of arsenic in drinking water and urine of women in Andes and Chaco.

| **Study groups** | ***n*** | **Arsenic in water (µg/L)** | ***n*** | **Urinary arsenic (µg/L)** |
| --- | --- | --- | --- | --- |
| **Andes** |  |  |  |  |
| San Antonio de los Cobres | 2 | 208 | 192 | 260 (20 – 1251) |
| Tolar Grande | 2 | 3.5 | 26 | 25 (10 – 121) |
| Salar de Pocitos | 2 | 72 | 5 | 73 (27 – 117) |
| Olacapato | 2 | 12 | 10 | 31 (14 – 56) |
| Santa Rosa de los Pastos Grandes | 1 | 31 | 15 | 71 (33 – 118) |
| Cobres | 1 | 3.5 | 13 | 47 (22 – 149) |
| Rosario de Lerma | 3 | 0.67 | 3 | 68 (64 – 147) |
|  |  |  |  |  |
| **Chaco** |  |  |  |  |
| Anta | 7 | 212 | 36 | 315 (70 – 1843) |
| El Rincón | 2 | 13 | 25 | 43 (17 – 97) |
| Los Rosales | 1 | 72 | 22 | 150 (60 – 877) |
| El Galpón | 7 | 4.7 | 42 | 20 (6.5 – 236) |
| Rivadavia | 2 | 243 | 26 | 346 (76 – 585) |
| La Unión | 1 | 23 | 12 | 53 (27 – 71) |
| Nueva Población | 1 | 983 | 6 | 1943 (623 – 2258) |

*The arsenic concentrations in water and urine were analyzed in our laboratory at Karolinska Institutet. The arsenic concentrations in water and urine for Andes population were previously reported in Concha et al. (Concha et al., 2010).

**Supplementary Material Table S2:** Characteristics of the Andes and Chaco study groups stratified for tertiles of U-As ^a^ (µg/L).

|  |  | ***Tertile 1*** |  |  | ***Tertile 2*** |  |  | ***Tertile 3*** |  |  |
| --- | --- | --- | --- | --- | --- | --- | --- | --- | --- | --- |
| **Variables** | ***n*** | **Median** | **5th-95th %** | ***n*** | **Median** | **5th-95th %** | ***n*** | **Median** | **5th-95th %** | ***p**- value** |
| Age (years) |  |  |  |  |  |  |  |  |  |  |
| *Andes* | 88 | 35 | 19-63 | 88 | 36 | 19-65 | 88 | 36 | 21-66 | 0.36 |
| *Chaco* | 57 | 43 | 20-64 | 56 | 37 | 18-74 | 56 | 41 | 16-64 | 0.83 |
| Sex (% women) |  |  |  |  |  |  |  |  |  |  |
| *Andes* | 88 |  | 86% | 88 |  | 92% | 88 |  | 90% | 0.46 |
| *Chaco* | 57 |  | 93% | 56 |  | 75% | 56 |  | 57% | <0.001 |
| BMI (kg/m^2^) |  |  |  |  |  |  |  |  |  |  |
| *Andes* | 88 | 25 | 19-35 | 88 | 26 | 21-35 | 88 | 25 | 19-35 | 0.96 |
| *Chaco* | 57 | 24 | 19-36 | 42 | 25 | 17-36 | 28 | 29 | 18-35 | 0.044 |
| Urinary arsenic (µg/L)^b^ | | |  |  |  |  |  |  |  |  |
| *Andes* | 88 | 53 | 15-124 | 88 | 196 | 142-266 | 88 | 368 | 275-660 | <0.001 |
| *Chaco* | 57 | 22 | 12-42 | 56 | 80 | 49-184 | 56 | 491 | 215-2016 | <0.001 |
| iAs in urine (%) | | |  |  |  |  |  |  |  |  |
| *Andes* | 88 | 9.1 | 3.7-23 | 88 | 11 | 4.6-24 | 88 | 12 | 4.6-24 | 0.026 |
| *Chaco* | 57 | 19 | 4.2-37 | 56 | 15 | 5.2-38 | 56 | 18 | 7.8-37 | 0.060 |
| MMA in urine (%) | | |  |  |  |  |  |  |  |  |
| *Andes* | 88 | 7.2 | 3.1-13 | 88 | 8.2 | 4.6-15 | 88 | 7.9 | 4.2-16 | 0.098 |
| *Chaco* | 57 | 9.1 | 2.5-16 | 56 | 9.1 | 3.1-18 | 56 | 15 | 6.4-24 | <0.001 |
| DMA in urine (%) | | |  |  |  |  |  |  |  |  |
| *Andes* | 88 | 83 | 68-92 | 88 | 80 | 64-90 | 88 | 79 | 65-90 | 0.017 |
| *Chaco* | 57 | 73 | 49-84 | 56 | 75 | 55-88 | 56 | 65 | 45-84 | 0.010 |
| mtDNAcn |  |  |  |  |  |  |  |  |  |  |
| *Andes* | 81 | 0.54 | 0.29-0.98 | 81 | 0.58 | 0.33-1.2 | 82 | 0.59 | 0.34-1.04 | 0.22 |
| *Chaco* | 50 | 0.74 | 0.42-1.30 | 55 | 0.80 | 0.52-1.8 | 54 | 0.97 | 0.42-1.8 | <0.001 |
| Telomere length |  |  |  |  |  |  |  |  |  |  |
| *Andes* | 69 | 0.43 | 0.30-0.56 | 70 | 0.42 | 0.29-0.58 | 69 | 0.44 | 0.31-0.63 | 0.079 |
| *Chaco* | 50 | 0.98 | 0.70-1.5 | 55 | 1.10 | 0.61-1.6 | 54 | 1.26 | 0.83-2.1 | <0.001 |

******p*-value is derived from Jonckheere-Terpstra trend test between the tertiles. ^a^U-As referred to as sum of urinary arsenic metabolites. ^b^adjusted to a mean specific gravity of 1.020.

**Supplementary Material Table S3:** General characteristics of the study participants with hyperkeratosis and remaining participants in Chaco.

|  | **Hyperkeratosis** |  | **Rest of Chaco** |  |
| --- | --- | --- | --- | --- |
| **Variables** | **Median (*n*=6)** | **Min to max** | **Median (*n*=153)** | **Min to max** |
| Urinary arsenic (µg/L) | 1943 | 623 to 2258 | 78 | 6.52 to 1843 |
| %iAs | 14 | 8.2 to 27 | 17 | 2.3 to 56 |
| %MMA | 21 | 18 to 24 | 10.2 | 0.75 to 27 |
| %DMA | 65 | 52 to 72 | 71 | 36 to 97 |
| mtDNAcn | 1.11 | 0.75 to 1.6 | 0.83 | 0.21 to 2.8 |
| Telomere length | 1.03 | 0.91 to 1.3 | 1.09 | 0.54 to 2.9 |

**Supplementary Material Table S4.** Multivariable regression analyses of the associations between U-As and log2-mtDNAcn stratified for arsenic metabolism efficiency (above and below median of fraction of each metabolite)^a^.

| **Study group** | **Median** | **% MMA** | ***n*** | **β_1_ (95% CI)^c^** | ***p-*value^c^** | ***p-*value^d^** |
| --- | --- | --- | --- | --- | --- | --- |
|  | **mtDNAcn^b^** |  |  |  |  |  |
| Andes | 0.56 | <7.9 | 122 | 0.017 (-0.034 to 0.069) | 0.50 | 0.48 |
|  | 0.58 | >7.9 | 122 | 0.030 (-0.035 to 0.095) | 0.37 |  |
| Chaco | 0.77 | <10 | 79 | 0.066 (-0.023 to 0.15) | 0.14 | 0.31 |
|  | 0.92 | >10 | 80 | 0.024 (0.0030 to 0.045) | 0.026 |  |
|  | **Telomere Length^b^** |  |  |  |  |  |
| Andes | 0.43 | <7.9 | 106 | 0.0048 (-0.0068 to 0.016) | 0.42 | 0.37 |
|  | 0.43 | >7.9 | 102 | 0.010 (0.0019 to 0.018) | 0.017 |  |
| Chaco | 1.06 | <10 | 79 | 0.050 (0.010 to 0.090) | 0.015 | 0.15 |
|  | 1.15 | >10 | 80 | 0.013 (-0.0024 to 0.028) | 0.098 |  |

^a^U-As referred to as sum of urinary arsenic metabolites. The number of individuals in Andes in each < and ≥ median split groups varied for telomere length as telomere length measurements were only available for individuals sampled in 2008.

^b^Presented as median relative values of mtDNAcn and telomere lenght.

^c^Log2-mtDNAcn / telomere length = α + β1 × U-As (per 100 µg/L) + β2 × age + β3 × gender

^d^*p*-value for interaction (β4) from the equation: Log2-mtDNAcn / telomere length = α + β1 × U-As (per 100 µg/L) + β2 × age + β3 × gender + β4 × (U-As × < and > median %iAs/%MMA/%DMA).
